# Supplementary material for: SyNDI: synchronous network data integration framework
Source: BMC Bioinformatics. 2018 Nov 6;19:403. doi: 10.1186/s12859-018-2426-5 (PMC6219086; doi:10.1186/s12859-018-2426-5)
Supplement: Supplementary file 5 — Files of the example presented in Additional file 4. This additional file is a zip package that contains all files used in Additional file 4. Detailed descriptions of each of these files are presented in a README file included in this zip package. (GZ 30411 kb) [file 12859_2018_2426_MOESM5_ESM.gz › SyNDITools/Meme2Fimo/meme_4.9.0_4.tar.gz!/meme_4.9.0/website/html/fimo-intro.html]

FIMO - Introduction


|  |  |
| --- | --- |
|  | MEME Suite The MEME Suite web application requires the use of JavaScript   Javascript doesn't seem to be available on your browser.  Introduction  FIMO is a tool for searching biological sequence databases for sequences that contain one or more known motifs.  The name FIMO stands for "Find Individual Motif Occurrences." A motif is a sequence pattern that occurs repeatedly in a group of related protein or DNA sequences. Motifs are represented as position-dependent scoring matrices that describe the score of each possible letter at each position in the pattern. Individual motifs may not contain gaps. Patterns with variable-length gaps must be split into two or more separate motifs before being submitted as input to FIMO. FIMO searches a sequence database for occurrences of user provided motifs, treating each motif independently. The program uses a dynamic programming algorithm to convert log-odds scores into p-values, assuming a zero-order background model. The p-values for each motif are then converted to q-values following the method of Benjamini and Hochberg (where "q-value" is defined as the minimal false discovery rate at which a given motif occurrence is deemed significant). The program reports all motif occurrences that receive p-values smaller than a specified threshold. Required FIMO Inputs  Three inputs must be provided on the FIMO web page:  1. An e-mail address where the notification of job completion can be sent.    You specify the e-mail address in the two text boxes labeled "e-mail address".    The e-mail address must be entered twice to reduce the amount of    undeliverable mail caused by typographic errors. 2. A MEME output file,    containing the descriptions of one or more motifs.    You can select a file to be uploaded from your computer by clicking on the    "Browse ..." button under the "Your motif file" label.    If you used the FIMO button on the MEME results page to get the FIMO web    page, then the motif file will automatically be filled in for you. 3. A sequence database to be searched. You can choose a sequence file to be    uploaded from your computer by clicking on the "Browse ..." button under the "Your    FASTA sequence file" label.    Alternatively, you can select one of the sequences databases listed in the    drop-down list box labeled "A supported database" .   Optional FIMO inputs  The FIMO web page accepts two optional inputs:  1. A threshold p-value. Motif occurrences with p-values above the threshold    will not be reported (defaults to `1e-4`). 2. A check-box directing that only the forward strand of the sequence    database will be scanned (defaults to unchecked).   The contents of the required and optional fields can be cleared by clicking on the "Clear Input" button. Submitting a FIMO job  After filling in the required and optional inputs on the FIMO web page, the job can be submitted for processing by clicking on the "Start Search" button. FIMO output  The result of submitting your job will be a summary page that briefly describes your job's input, or reports any critical errors. If there are no errors, then the page will also contain a link to your job's results. The results page will report the full command that was run and will include links to the FIMO output, or to any error messages that were generated. The FIMO output will be a list of matches to the input motifs, sorted by p-value. This will be provided in several formats:  - HTML (example) - XML using the CisML schema   (example) - GFF   (example) - Plain text (example) |
